# Relationship between physical activity and cerebral white matter hyperintensity volumes in older adults with depressive symptoms and mild memory impairment: a cross-sectional study

**DOI:** 10.3389/fnagi.2024.1337397

**Published:** 2024-02-13

**Authors:** Shotaro Otsuka, Kiyoshi Kikuchi, Yasufumi Takeshita, Seiya Takada, Akira Tani, Harutoshi Sakakima, Ikuro Maruyama, Hyuma Makizako

**Affiliations:** ^1^Department of Laboratory and Vascular Medicine, Graduate School of Medical and Dental Sciences, Kagoshima University, Kagoshima, Japan; ^2^Division of Brain Science, Department of Physiology, Kurume University School of Medicine, Kurume, Japan; ^3^Department of Neurosurgery, Kurume University School of Medicine, Kurume, Japan; ^4^Department of Physical Therapy, School of Health Sciences, Faculty of Medicine, Kagoshima University, Kagoshima, Japan

**Keywords:** aging, depressive symptoms, memory problems, physical activity, interleukin 6 (IL-6)

## Abstract

**Introduction:**

Cerebral white matter hyperintensities (WMHs) are commonly found in the aging brain and have been implicated in the initiation and severity of many central nervous system diseases. Furthermore, an increased WMH volume indicates reduced brain health in older adults. This study investigated the association between WMH volume and physical activity in older adults with depressive symptoms (DS) and mild memory impairment (MMI). Factors associated with the WMH volume were also investigated.

**Methods:**

A total of 57 individuals aged over 65 years with DS and MMI were included in this study. The participants underwent magnetic resonance imaging to quantify WMH volumes. After WMH volume was accumulated, normalized to the total intracranial volume (TIV), the percentage of WMH volume was calculated. In addition, all participants wore a triaxial accelerometer for 2 weeks, and the average daily physical activity and number of steps were measured. The levels of blood biomarkers including cortisol, interleukin-6 (IL-6), brain-derived insulin-like growth factor-1, and brain-derived neurotrophic factor were measured. Motor and cognitive functions were also assessed.

**Results:**

Faster maximum walking speed and longer time spent engaged in moderate physical activity were associated with a smaller percent of WMH volume, whereas higher serum IL-6 levels were associated with a larger percent of WMH volume. The number of steps per day, time spent engaged in low levels of physical activity, cognitive function, and all other measured biomarkers were not significantly associated with percent of WMH volume.

**Discussion:**

Higher blood inflammatory cytokine levels, shorter duration of moderate physical activity, and lower maximum walking speed were associated with a higher percent of WMH volume. Our results provide useful information for maintaining brain health in older adults at a high risk of developing dementia and may contribute to the development of preventive medicine for brain health.

## 1 Introduction

Magnetic resonance imaging (MRI) often reveals cerebral white matter hyperintensities (WMHs) in older adults. WMHs are a primary manifestation of white matter damage, including damage to small blood vessels, amyloid angiopathy, ischemic hypoxic damage, altered blood–brain barrier properties, demyelination, and axonal loss (Fernando et al., 2006; Black et al., 2009; Gouw et al., 2011; Han et al., 2019). WMHs are common in older adults and their volume increases over time (Erten-Lyons et al., 2013; Promjunyakul et al., 2015). In addition, numerous risk factors for cardiovascular diseases such as smoking and hypertension increase the prevalence of WMHs (Staals et al., 2014). WMHs can be anatomically divided into deep subcortical WMHs (DSWMHs) and periventricular hyperintensities (PVHs) based on their location and appearance. The involvement of DSWMHs in mood disorders has been reported (De Groot et al., 2000), whereas PVHs are associated with cognitive memory impairment (Bolandzadeh et al., 2012). Furthermore, larger WMH volumes are associated with a higher risk of Alzheimer’s disease (Gaubert et al., 2021), stroke (Kuller et al., 2004; Wen and Sachdev, 2004), motor dysfunction (Silbert et al., 2008), and mortality (Debette et al., 2010). However, there are currently no specific preventive measures or treatments for reducing WMH volume. Thus, to improve the early detection of WMHs, it is crucial to identify risk factors.

Several studies have documented the neuroprotective effects of physical activity against Alzheimer’s disease, stroke, lacunar infarction, and traumatic head injuries. For example, a study of 55 older adults with mild memory impairment suggested that 16 weeks of aerobic exercise was effective in increasing neurotrophic factors, decreasing certain inflammatory cytokines, and promoting neurocognitive function (Tsai et al., 2019). Moreover, animal studies have reported that treadmill training for 3 weeks before stroke onset increases neuroprotective factor expression and has neuroprotective effects, resulting in reduced infarct volume and motor sensory deficits (Otsuka et al., 2016, 2019). In addition, physical activity has beneficial effects on WMHs. A study showed that higher self-rated physical activity at baseline was linked to smaller WMH volume in the deep and periventricular regions after 3 years (Gow et al., 2012). Another study showed a relationship between increased self-rated physical activity at baseline and WMH (periventricular and deep) progression at 5 years in a cognitively intact group only (Bolandzadeh et al., 2012). However, another 5-year follow-up study of 180 people aged ≥65 years showed no association between physical activity and progression of WMH (Podewils et al., 2007). Notably, most previous studies used self-administered questionnaires to investigate physical activity. Self-reported physical activity is vulnerable to bias caused by inaccurate memory and other factors that may lead to measurement errors, preventing detailed physical activity data from being obtained (Prince et al., 2008). Direct measurement of physical activity using pedometers and triaxial accelerometers may eliminate these errors. In a previous study, physical activity was measured over a short period of 1 week to assess its association with WMH volume (Bangen et al., 2023). Nevertheless, there are limited studies on the use of triaxial accelerometers to measure physical activity and how it relates to WMH volume.

WMHs are associated with several risk factors including aging, hypertension, smoking, body mass index, and the presence of heart disease or diabetes (Johnson et al., 2019; Garfield et al., 2021). Additionally, WMH volume may be influenced by neurotrophic factors that confer neuroprotective effects and inflammatory cytokines involved in various cerebrovascular disorders. Although individual serum biomarkers and certain risk factors related to brain structural changes and WMH volumes are not well established, the identification of WMH-related serum biomarkers will aid in the development of interventions aimed at maintaining brain health in older adults.

Depressive symptoms (DS) and memory loss are common in older adults. Furthermore, older adults with both DS and memory loss are more likely to develop dementia (Paterniti et al., 2002; Barnes and Yaffe, 2011; Yaffe et al., 2011), which is likely to also be associated with increased WMH volumes. However, to date, no studies have investigated the relationship between WMH volume and daily physical activity in older adults with DS and mild memory impairment (MMI). Previous studies examining WMHs have focused on healthy older adults and those with dementia. However, no studies have investigated older adults presenting with symptoms of depressive tendencies and MMI associated with the development of dementia. Depressive tendencies and MMI may improve, and investigating the relationship between white matter lesion volume and physical activity level may provide important information for health management of older adults. Therefore, the present study focused on the association between white matter lesion volume and physical activity volume in community-dwelling older adults (aged ≥65 years) with DS and MMI, who are at a high risk of developing dementia. We also aimed to identify factors (blood biomarkers, mobility, and memory function) that may be associated with WMH and physical activity volumes.

## 2 Materials and methods

### 2.1 Study design and participants

**Figure 1:**
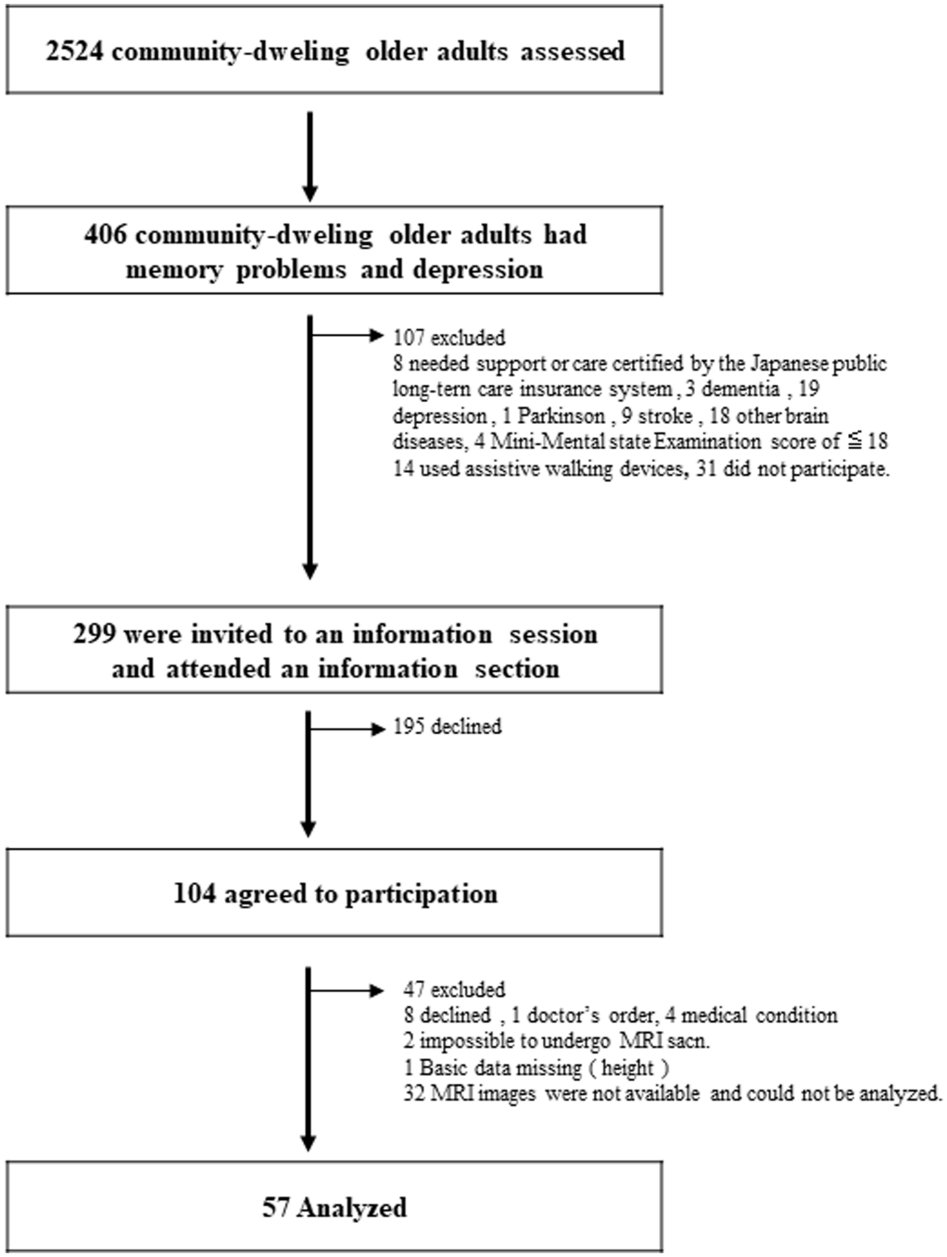
Flow diagram depicting the participant screening process.

This study analyzed the baseline data from previous community-based, single-blind, randomized controlled experiments conducted in Japan (Makizako et al., 2015a, 2019). The clinical investigation was preregistered with the clinical trial registration of the University Hospital Medical Information Network (000018547). This interventional study was conducted between February 2015 and October 2015 in Obu, Japan. In all, 2,524 older adults aged ≥65 years were enrolled in this study. From this pool, 406 patients with DS and MMI were selected (Figure 1). The inclusion criteria were as follows: (1) individuals aged ≥65 years residing in a community on their own, (2) exhibiting depressive symptoms with a Geriatric Depression Scale-15 (GDS-15) score of at least 529, and (3) memory impairment, as evidenced by an age-adjusted word list memory score > 1.0 standard deviation (SD) below the criteria threshold, which may be either subjective memory impairment or objectively measured moderate memory loss. The following were the exclusion requirements: (1) obtaining assistance or care as attested by the government-run Japanese long-term care insurance system, (2) a Mini-Mental State Examination (MMSE) score of <18 or a diagnosis of dementia, (3) a history of a severe mental illness or another neurological or musculoskeletal condition, (4) any disability that affects basic activities of daily living, (5) inability to undertake tests of cognitive performance, (6) contraindications for physical exercise, and (7) daily use of walking aids. Finally, data from 57 older adults were analyzed (see Figure 1 for details of the excluded participants). All 57 patients underwent MRI for WMH analysis. Prior to enrolment, the participants provided written informed consent. The study protocol was approved by the Ethics Committee of the National Center for Geriatrics and Gerontology in Japan (#839).

### 2.2 Setting

The investigation was conducted in the neighborhood of Nagoya, Japan, in Obu City. Since 2011, we have been conducting observational research in this area, including in-person interviews and evaluations of physical and mental capacity (Shimada et al., 2013). Data collection and participant screening were performed at a community center.

### 2.3 Data collection

Between February 2015 and October 2015, participants were required to participate in 2 h of baseline data collection, wherein they were assessed for general health (including blood tests), physical ability, and cognitive function. In addition, the National Center for Geriatrics and Gerontology collected MRI data to study WMHs in the brain. In the present study, we selected measures reported to be associated with WMH volume. Physical performance was assessed using a grip strength test to measure muscle strength, up-and-go test with timing to assess balance, and maximal gait speed test to measure general walking ability (Pinter et al., 2018). Grip strength, timed up-and-go, and gait speed tests are easy and simple to administer in community settings and are powerful predictors of health outcomes (Rantanen, 2003). The MMSE and word-list memory tasks (Makizako et al., 2013) were used to assess cognitive function. The MMSE is a 10-min test that evaluates general cognitive and memory capabilities. This examination includes 30 questions assessing memory, orientation, attention, math, language, and vision. The word-list memory challenge involved both delayed and quick recall of a target list of 10 words presented for 2 s. Next, 30 words (10 target words and 20 interference phrases) were proposed, and the participants were prompted to immediately select the 10 target words. This procedure was repeated thrice. The total score is determined by calculating the average number of correct responses. In addition, for a “delayed” score, the participants had to recollect 10 target phrases after around 20 min. The GDS-15 was used to assess depressive symptoms. A score of <5 on the 15-item GDS-15 was used to identify the presence of clinical depression symptoms (van Marwijk et al., 1995).

During the 2 weeks after baseline measurements, physical activity was continually measured, as detailed in a previous study (Makizako et al., 2015b) using a 3-axis accelerometer (modified HJA-350IT, Active Style Pro, Omron Healthcare Co., Ltd., Kyoto, Japan) (Oshima et al., 2010). For 2 weeks, the participants were instructed to wear an accelerometer on a waistband. Metabolic equivalents (multiples of the resting metabolic rate) were used to express the output. The non-wearing time was defined as the time at which the accelerometer data were not recorded for a period longer than a minute. We defined a low level of physical activity as 1.5–2.9 metabolic equivalents and a moderate level as 3.0–5.9 metabolic equivalents, as per a previous study (Pate et al., 1995). Every 4 s, the accelerometer counted the steps taken and calculated the amount of physical activity based on the pattern of the accelerometer signal (Oshima et al., 2010; Ohkawara et al., 2011; Moniruzzaman et al., 2020). Prior to calculating the parameters, the activity time data were examined using a computer program; if there was a continuous interval during which no body movements were recorded, it indicated a period of time during which the monitor was removed (e.g., while bathing or sleeping). Participants who did not record ≥75 percent of daytime activities (i.e., from 6 a.m. to 6 p.m.) for at least 7 days throughout the 2-week period were excluded from the study. To prevent the participants from monitoring their step counts and values to gauge their typical daily activities over the 2-week period, the accelerometer displays were blinded.

The following biomarker concentrations were measured in blood samples related to WMH volume: cortisol (Cox et al., 2015), hemoglobin A1c (HbA1c) (De Havenon et al., 2019), interleukin (IL)-6 (Satizabal et al., 2012), insulin-like growth factor-1 (IGF-1) (Wittfeld et al., 2022), highest-density lipoprotein (HDL) cholesterol (Johnson et al., 2019), total cholesterol (Williams et al., 2013), and brain-derived neurotrophic factor (BDNF) (Dalby et al., 2013). Serum IL-6 levels were measured using a validated chemiluminescent enzyme immunoassay (Shiga et al., 2016), serum IGF-1 levels were quantified using an IGF-1 immunoradiometric assay (Daiichi; TFB Inc., Tokyo, Japan) (Doi et al., 2015), and serum BDNF levels were assessed using a Quantizing Human Kit (R&D Systems, Inc., Minneapolis, MN, United States). Assays were performed at SRL Tokyo Laboratories Inc., (Tokyo, Japan) (Suzuki et al., 2013).

### 2.4 Measurements of WMH volumes

WMH volume was measured as previously described (Syaifullah et al., 2020, 2021). A 3 T MRI scanner (TIM Trio, Siemens, Munich, Germany) was used to scan the entire brain. Participants who could not be accurately scanned in a single scan were excluded. Prior to voxel-based morphometric analysis, the MRI images were converted into files that conformed to the Neuroimaging Informatics Technology Initiative. Scan images were edited by Brain Anatomical Analysis Using Diffeomorphic Deformation (BAAD version 4.3; accessible at http://www.shiga-med.ac.jp/hqbioph/BAAD (English)/BAAD.html). WMHs were divided into DSWHMs and PVHs, and their volumes were calculated from fluid-attenuated inversion recovery (FLAIR) images using BAAD software.

The brain MRI acquisition protocol has been described in detail in previous studies (Vellas et al., 2014; Moon et al., 2017). Briefly, three-dimensional (3D) volume acquisition of a T1-weighted gradient-echo sequence (inversion time [TI], 800 ms; repetition time [TR], 1800 ms; echo time [TE], 1.98 ms; slice thickness, 1.1 mm) was acquired to create a gapless series of thin sagittal sections using the gradient echo sequence with quick magnetization preparation acquisition (TI, 800 ms; TR, 1800 ms; TE, 1.98 ms; slice thickness, 1.1 mm). Axial FLAIR images (TI, 2500 ms; TR, 9000 ms; TE, 100 ms; slice thickness, 5.0 mm) and T2-weighted spin-echo images (TR, 4200 ms; TE, 89.0 ms; slice thickness, 5.0 mm) were acquired.

**Figure 2:**
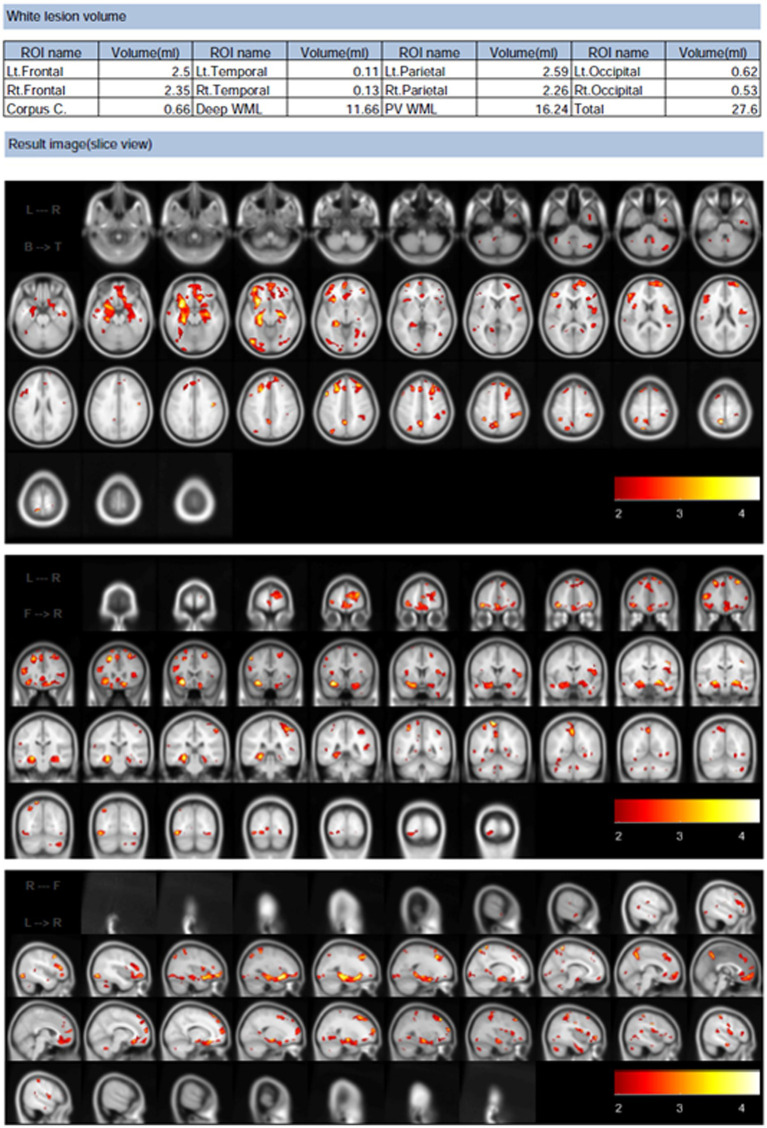
Example of white matter hyperintensities volume analysis results calculated using Brain Anatomical Analysis Using Diffeomorphic Deformation software. These results are of the horizontal, coronal, and sagittal planes analysis, wherein white matter lesions are brightened in red and other colors.

All parameters in the preprocessing steps were determined using default settings. Briefly, images were set around the anterior commissure–posterior commissure line and resampled with a 1-mm^3^ voxel size. Computational Anatomy Toolbox 12 (Structural Brain Mapping Group, Jena University Hospital, Jena, Germany) was used for tissue segmentation and intensity nonuniformity correction. Because WMHs exert signal intensities, such as gray matter, on T1-weighted images, we conducted automated WMH correction of the FLAIR images using the Lesion Segmentation Toolbox software (Schmidt et al., 2012). The WMH was masked using the average signal intensity of the surrounding normal white matter prior to gray and white matter segmentation. Total intracranial volume in cm^3^ and WMH volume in cm^3^ were measured using MRI. The tissue volumes provided by the SPM5 toolbox^1^ were used to calculate the total intracranial volume from the 3D T1-weighted data. Using software that automatically separates WMH from FLAIR and 3D T1-weighted images, WMH volumes were calculated from MRI scans using white matter hyperintensity (Samaille et al., 2012). In contrast to the intensity-based approach, this method relies on the contrast. The typical preprocessing steps were as follows: tissue information was derived from the 3D T1-weighted images and then registered to the FLAIR images to correct for intensity inhomogeneities. The WMH contrast in the FLAIR image was then improved using a nonlinear diffusion framework to produce a piecewise constant image. Finally, a pertinent region was selected based on the location of the tissue profile data collected during the preprocessing stage. Consequently, we calculated WMH volume (mL) from the MRI images (Figure 2). After WMH volume accumulation was normalized to the total intracranial volume (TIV), the percentage of WMH volume (percent of WMH) was calculated.

### 2.5 Statistical analysis

SPSS 27.0 for Windows (SPSS, Inc., Chicago, IL, United States) was used for the statistical analyses. The data has been presented as means ± SDs. Multivariable linear regression was used to examine (1) the correlations between percent of WMH (DSWMH and PVH) volumes and body mass index, motor function assessment (grip strength, up-and-go time, and maximum walking speed), DS, and cognitive function (i.e., GDS-15, MMSE, and word list memory [immediate and delayed]); (2) the relationship between percent of WMH volumes and blood biomarkers (i.e., cortisol, HDL and total cholesterol, IL-6, IGF-1, BDNF, and HbA1c); (3) the relationship between percent of WMH volumes and physical activity (i.e., number of steps, time spent doing a low level of activity, and time spent doing a moderate level of activity); (4) the relationship between IL-6 level and times spent doing a moderate level of activity and maximum walking speed.

All regression models were evaluated using the Pearson’s correlation coefficient (*r*). Furthermore, age and sex were adjusted (*β*). Differences were considered statistically significant at *p* < 0.05.

## 3 Results

### 3.1 Relationships between percent of WMH volumes and participant characteristics

In this study, a secondary examination of data from a previous study was conducted (Makizako et al., 2019). Table 1 presents information on the participant characteristics. The mean age of the participants was 73.03 years, and 42.1% (*n* = 24) were female. Additionally, 43.9% (*n* = 25) of the participants had hypertension and 24.6% (*n* = 14) had diabetes. The mean (SD) TIV of the participants was 1505.57 mL (133.04 mL). The DSWMH volume was 0.67% (0.12%) and PVH volume was 0.59% (0.45%). Table 2 shows the relationships between the 2 % of WMH volumes (DSWMH and PVH) and participant characteristics (body mass index, physical performance, GDS-15, and general cognitive function test), which were evaluated using the Pearson’s correlation coefficient (*r*). Furthermore, age and sex (*β*) were adjusted. Body mass index, GDS-15, and general cognitive function tests were not significantly correlated with percent of WMH volumes. Regarding physical performance, no significant correlations were found between grip strength, time up and go, and percent WMH volumes. However, a significant correlation was found between maximal walking speed and percent of DSWMH volume (*β* = −0.318, *p* = 0.018) or percent of PVH volume (*β* = −0.328, *p* = 0.015). It was clear that the higher the maximum walking speed, the lower the percent of WMH volumes.

**Table 1:** Participant characteristics (*n* = 57).

*Sex, n (%)*	
Male	33 (57.9)
Female	24 (42.1)
Age (years)	73.1 ± 5.6
Height (cm)	159.1 ± 9.4
Weight (kg)	58.9 ± 10.6
Body mass index, kg/m^2^	22.9 ± 3.7
*Illness incidences (%)*	
Hypertension	25 (43.9)
Heart disease	8 (14.0)
Diabetes mellitus	14 (24.6)
*Physical performance*	
Grip strength, kg	28.5 ± 8.5
Timed up and Go, s	8.3 ± 1.2
Maximum walking speed, m/s	1.6 ± 0.2
*Depressive symptoms*	
GDS-15, score	6.4 ± 1.9
*General cognitive function*	
MMSE, score	27.9 ± 2.1
Word list memory (immediately), score	7.9 ± 1.5
Word list memory (delay), score	4.9 ± 2.1
*Brain volumes from magnetic resonance imaging*	
DSWMH, mL	10.1 ± 10.9
PVH, mL	8.9 ± 6.9

Data are presented as *n* (%) or mean ± standard deviation. *n*, number; GDS-15, Geriatric Depression Scale-15; MMSE, Mini-Mental State Examination; DSWMH, deep subcortical white matter hyperintensity; PVH, periventricular white matter hyperintensity.

**Table 2:** Relationships between the percent of white matter hyperintensity volumes and participant characteristics.

	DSWMH				PVH			
Primary outcomes	Simple-correlation (*r*)	*p* value	Age and sex-adjusted (*β*)	*p* value	Simple-correlation (*r*)	*p* value	Age and sex-adjusted (*β*)	*p* value
Body mass index, kg/m^2^	−0.076	0.574	−0.066	0.635	−0.103	0.445	−0.096	0.485
*Physical performance*								
Grip strength, kg	0.137	0.309	0.092	0.504	0.039	0.773	−0.034	0.807
Timed up and Go, s	0.219	0.102	0.231	0.089	0.216	0.107	0.226	0.097
Maximum walking speed, m/s	−0.277	0.037*	−0.318	0.018*	−0.295	0.026*	−0.328	0.015*
*Depressive symptoms*								
GDS-15, score	−0.218	0.104	−0.228	0.094	−0.219	0.102	−0.226	0.097
*General cognitive function*								
MMSE, score	−0.181	0.801	0.065	0.636	−0.052	0.701	−0.034	0.803
Word list memory (immediately), score	−0.023	0.864	0.022	0.875	−0.127	0.348	−0.105	0.444
Word list memory (delay), score	−0.042	0.759	0.012	0.928	−0.152	0.259	−0.132	0.335

^*^p < 0.05, ^**^p < 0.01. DSWMH, deep subcortical white matter hyperintensity; PVH, periventricular white matter hyperintensity; GDS-15, Geriatric Depression Scale-15; MMSE, Mini-Mental State Examination.

### 3.2 Serum IL-6 and moderate physical activity are associated with percent of WMH volumes

Table 3 shows the relationships between the 2 % of WMH volume (DSWMH and PVH) and blood biomarkers. Table 4 shows the relationships between the 2% of WMH volumes (DSWMH and PVH) and physical activity levels, which were evaluated using the Pearson’s correlation coefficient (*r*). Age and sex were adjusted. Neither cortisol nor HbA1c were significantly correlated with DSWMH (*β* = 0.255, *p* = 0.06 and *β* = 0.168, *p* = 0.22, respectively) or PVH volume (*β* = 0.255, *p* = 0.06 and *β* = 0.181, *p* = 0.187, respectively). Additionally, HDL cholesterol, total cholesterol, IGF-1, and BDNF levels were not significantly correlated with WMH volume. In contrast, serum IL-6 levels significantly correlated with both percent of DSWMH (*β* = 0.305, *p* = 0.023) and PVH volumes (*β* = 0.353, *p* = 0.008), where higher serum IL-6 levels were associated with higher percent of WMH volumes. On average, participants completed 5732.1 ± 2829.8 steps/day (mean ± SD). There was no significant correlation between percent of DSWMH (*β* = −0.264, *p* = 0.136) or PVH (*β* = − 0.183, *p* = 0.181) volumes and the number of steps per day. In addition, time required to perform a low level of activity was not significantly correlated with percent of DSWMH (*β* = −0.033, *p* = 0.811) or PVH (*β* = −0.046, *p* = 0.736) volumes. However, there was a significant correlation between time required to perform an activity of moderate level and DSWMH (*β* = −0.389, *p* = 0.004) and PVH volumes (*β* = −0.362, *p* = 0.007), where higher time required to perform an activity of moderate level was associated with lower percent of WMH volumes.

**Table 3:** Relationships between the percent of white matter hyperintensity volumes and blood biomarkers.

		DSWMH				PVH			
Primary outcomes		Simple-correlation (*r*)	*p* value	Age and sex-adjusted (*β*)	*p* value	Simple-correlation (*r*)	*p* value	Age and sex-adjusted (*β*)	*p* value
Cortisol, μg/dL	10.4 ± 5.0	0.255	0.055	0.255	0.06	0.250	0.061	0.255	0.06
HDL cholesterol, mg/dL	60.1 ± 15.6	0.056	0.68	0.081	0.559	0.16	0.235	0.179	0.19
Total cholesterol, mg/dL	201.9 ± 3.0	−0.206	0.124	−0.189	0.167	−0.125	0.355	−0.058	0.676
IL-6, pg./mL	2.3 ± 1.8	0.318	0.016*	0.305	0.023*	0.359	0.006**	0.353	0.008**
IGF-1, ng/dL	97.4 ± 25.9	0.103	0.447	0.099	0.474	0.13	0.353	0.134	0.33
BDNF, ng/dL	18479.3 ± 8476.6	−0.156	0.246	−0.153	0.266	−0.208	0.121	−0.206	0.132
HbA1c, %	5.9 ± 0.6	0.164	0.223	0.168	0.22	0.178	0.186	0.181	0.187

Data are presented as means ± standard deviations. ^*^p < 0.05, ^**^p < 0.01. DSWMH, deep subcortical white matter hyperintensity; PVH, periventricular white matter hyperintensity; HDL, high-density lipoprotein; IL-6, interleukin-6; IGF-1, brain-derived insulin-like growth factor-1; BDNF, brain-derived neurotrophic factor; HbA1c, hemoglobin A1c.

**Table 4:** Relationships between the percent of white matter hyperintensity volumes and physical activity.

		DSWMH				PVH			
Primary outcomes		Simple-correlation (*r*)	*p* value	Age and sex-adjusted (*β*)	*p* value	Simple-correlation (*r*)	*p* value	Age and sex-adjusted (*β*)	*p* value
Steps, steps/day	5732.1 ± 2829.8	−0.239	0.074	−0.264	0.136	−0.236	0.077	−0.183	0.181
Low activity time, min/day	301.9 ± 93.5	−0.081	0.55	−0.033	0.811	−0.077	0.569	−0.046	0.736
Moderate activity time, min/day	49.8 ± 32.3	−0.384	0.003^**^	−0.381	0.004^**^	−0.359	0.006^**^	−0.362	0.007^**^

Data are presented as means ± standard deviations. ^*^p < 0.05, ^**^p < 0.01. DSWMH, deep subcortical white matter hyperintensity; PVH, periventricular white matter hyperintensity.

### 3.3 Association between serum IL-6 level and time required to perform an activity of moderate level and maximum walking speed

We examined whether serum IL-6 levels were associated with duration of moderate activity and maximal walking speed. Moderate activity time showed a significant inverse correlation with serum IL-6 levels (*r* = −0.287, *p* = 0.03). However, when adjusted for age and sex, the correlation was not significant (*β* = −0.232, *p* = 0.088). Maximal walking speed was not correlated with serum IL-6 level (*r* = −0.006, *p* = 0.964; *β* = −0.018, *p* = 0.899).

## 4 Discussion

The purpose of this study was to examine the association between WMH volume and physical activity in older adults with DS and MMI and to identify factors associated with WMH volume. According to our results, longer periods of moderately intense physical activity and faster maximal walking speeds were associated with decreased percent of WMH volume. In addition, a higher percent of WMH volume were associated with higher blood IL-6 levels.

Longer daily physical activity above a certain intensity may affect the WMH volume. Previous research has documented that lifestyle factors, such as dietary habits and physical exercise, are associated with both cortical thickness (Reiter et al., 2015; Lee et al., 2016) and WMH volume (Mosconi et al., 2014). We focused on the amount of physical activity and the number of steps taken per day and investigated how these factors relate to WMH volumes. In a 5-year follow-up study of 680 men, there was a negative correlation between the number of steps taken at baseline and WMH volume after 5 years, with a minimum of 8,150 steps per day required to reduce WMH volume (Moniruzzaman et al., 2020). The number of daily steps taken by the participants in the present study was 5732.0 ± 2829.8 (mean ± SD) and was not correlated with percent of WMH volumes. We speculate that these discrepant findings can be attributed to the relatively small number of steps taken in our investigation. A previous study on the relationship between physical activity and WMH volume in 88 healthy individuals aged 60–78 years reported that moderate or high levels of physical activity were more strongly associated with lower WMH volume than light physical activity (Burzynska et al., 2014). Several studies have found a link between increased physical activity intensity and decreased WMH volume (Gow et al., 2012; Sen et al., 2012; Tseng et al., 2013). In addition, a cross-sectional study of adults aged ≥65 years reported that a lower maximum walking speed at baseline is associated with WMH volume (Soumaré et al., 2009). Similar results were obtained in our study, suggesting that maximal walking speed is an important factor in the assessment of motor function.

The expression of BDNF and IGF-1 in blood is enhanced by physical activity (Cassilhas et al., 2010; Jeon and Ha, 2017). Based on the results showing that moderate physical activity is associated with percent of WMH volumes, we hypothesized that BDNF and IGF-1 may also be associated with WMH volume. These two neurotrophic factors have been documented to play important roles in neurogenesis, angiogenesis, and survival (McAllister et al., 1995; Horch, 2004; Åberg et al., 2006; Hung et al., 2007). According to Pikula et al. (2013), higher levels of serum BDNF are associated with lower WMH volumes. Furthermore, reduced WMHs in the prefrontal cortex are associated with higher plasma BDNF expression levels (Dalby et al., 2013). Meta-analyses have reported that the higher the IGF-1 concentration, the lower is the WMH volume (Wittfeld et al., 2022). Moreover, an earlier study that followed 757 older adults for 10 years reported that lower serum IGF-1 levels were associated with a higher incidence of stroke (Saber et al., 2017). In this study, we did not observe any correlation between blood BDNF and IGF-1 concentrations or WMH volume. Shimada et al. (2014) reported that older adults with DS have lower serum BDNF levels than cognitively normal older adults (Shimada et al., 2014). Moreover, reduced serum IGF-1 levels are associated with mild dementia (Doi et al., 2015). Our patient had symptoms of DS and MMI, which may have adversely affected the expression of neurotrophic factors (BDNF and IGF) and reduced their association with WMH volume. New insights can be gained by investigating the relationship between neurotrophic factors other than BDNF and IGF and WMH volume, which were examined in the present study.

Our study revealed that IL-6 is an important blood biomarker that may be associated with WMH volume in older adults with DS and MMI. WMHs are often observed in older adults and are associated with a higher risk of overall mortality, stroke, and cognitive and physical deterioration (Debette and Markus, 2010; Silbert et al., 2012). Inflammatory mediators are strongly associated with WMHs (Torres et al., 2015). An earlier cross-sectional study that included healthy older people aged 65–80 years found a correlation between higher concentrations of IL-6 and C-reactive protein and higher WMH volume (Satizabal et al., 2012). Conversely, among older persons without dementia, higher IL-6 levels are linked to reduced brain volume, but not WMH volume (Gu et al., 2017). Given that our participants were older adults with DS and MMI who lived in their communities, they may have had higher serum IL-6 levels than cognitively normal older adults. Indeed, previous studies have reported that patients with DS have higher expression of inflammatory cytokines and IL-6 than individuals without such symptoms (Ryan et al., 2017). Thus, IL-6 levels may not be significantly correlated with WMH volume in cognitively normal older adults. Nevertheless, multiple earlier studies have found a link between a high inflammatory state and high WMH volume (Schuitemaker et al., 2009; Lampe et al., 2019).

A longer time spent performing physical activity of moderate levels was not associated with lower serum IL-6 levels. Previous studies in primary care patients reported that moderate physical activity decreases the expression of serum IL-6 (Rethorst et al., 2011). In addition, Paolucci et al. reported that exercise of more than moderate intensity is required to exert anti-inflammatory effects (Paolucci et al., 2018). Thus, we hypothesized that the time spent performing moderate-intensity physical activity affects IL-6 expression and reduces WMH volume. However, our results did not support this hypothesis. The fact that our study participants had symptoms of depression and were less physically active than healthy adults of similar age may have contributed to this discrepancy. Future comparisons of activity levels among healthy older adults may reveal a more detailed relationship between these two factors, and how they relate to WMH volume.

In this study, we divided WMH into DSWMH and PVH and examined the associated factors at these different sites. An association between increased PVH volume and greater cognitive impairment has been observed (Kim et al., 2008), whereas in our study, percent of PVH and DSWMH volumes were not associated with memory impairments. Furthermore, DSWMHs have been reported to be involved in mood disorders (De Groot et al., 2000), which was not observed in the present study. These discrepant findings may be attributed to differences in the assessment methods and symptom severity. Although the participants in our study were older than 65 years and had DS and MMI, their mood and memory impairments were likely not associated with the percent of WMH volumes because their impairments were relatively mild. Furthermore, higher serum IL-6 levels were associated with higher percent of DSWMH and PVH volumes, whereas no other biomarkers were associated with the percent of DSWMH or PVH volumes. The longer the time spent on moderate or high levels of physical activity, the smaller the percent of DSWMH and PVH volumes. Thus, prolonged physical activity may reduce WMH volumes in all brain regions.

A limitation of this study is its cross-sectional design, as causal effects could not be explored. In addition, the sample size was relatively small (*n* = 57). WMH volumes could not be measured using MRI in several participants, which reduced the number of samples available for analysis. In addition, our results were based only on community-dwelling older adults with DS or MMI. Healthy older adults may have different factors that affect WMH volume. We were unable to determine a detailed relationship between physical activity and WMH volume, which may have been made possible by comparing the activity levels obtained in our study with those of healthy older adults. Furthermore, smoking history, a factor that influences the WMH volume, was not included in the survey. However, none of the participants had a history of chronic obstructive pulmonary disease. Thus, the results were unlikely to have been affected by this omission. Further studies with larger sample sizes are required to provide more detailed insight.

In summary, the present study aimed to examine the association between WMH volume and physical activity in high-risk community-dwelling older adults aged ≥65 years with DS and MMI and to identify the factors associated with WMH volume. We analyzed the MRI images, classified the WMHs into two categories (DSWMH and PVH), and calculated their volumes. Elevated levels of inflammatory cytokines in blood are associated with higher percent of DSWMH and PVH volumes. In contrast, faster maximum walking speed and more time spent performing a moderate level of physical activity correlated with smaller percent of DSWMH and PVH volumes. Given that high WMH volumes are associated with adverse effects on the brain, early detection and treatment are vital. Our results provide valuable information for maintaining brain health in older adults with DS and MMI, and may contribute to the development of basic medicine for brain health.

## Data availability statement

The raw data supporting the conclusions of this article will be made available by the authors, without undue reservation.

## Ethics statement

The studies involving humans were approved by the clinical investigation was pre-registered with the clinical trial registration of the University Hospital Medical Information Network (000018547). The studies were conducted in accordance with the local legislation and institutional requirements. The participants provided their written informed consent to participate in this study.

## Author contributions

SO: Formal analysis, Software, Writing – original draft. KK: Methodology, Resources, Writing – review & editing. YT: Formal analysis, Writing – review & editing. ST: Formal analysis, Writing – review & editing. AT: Methodology, Writing – review & editing. HS: Project administration, Resources, Writing – review & editing. IM: Methodology, Writing – review & editing. HM: Funding acquisition, Methodology, Project administration, Resources, Writing – original draft.

## Footnotes

^1^ http://www.fil.ion.ucl.ac.uk/spm
